# Supplementary material for: Unified Focal loss: Generalising Dice and cross entropy-based losses to handle class imbalanced medical image segmentation
Source: Comput Med Imaging Graph. 2022 Jan;95:102026. doi: 10.1016/j.compmedimag.2021.102026 (PMC8785124; doi:10.1016/j.compmedimag.2021.102026)
Supplement: Supplementary file 1 — Supplementary material [file mmc1.pdf]

Table 1: Hyperparameter tuning using the asymmetric variant of the Unified Focal loss on the BUS2017 dataset, confirming the default values chosen for δ and λ. Values are reported from five-fold cross validation and in the form mean $\pm$ 95% confidence intervals. Numbers in boldface denote the highest values for each metric.

$\gamma = 0$, $\lambda = 0.5$				$\delta = 0.6$, $\gamma = 0.2$			
δ	DSC	Precision	Recall	λ	DSC	Precision	Recall
0.1	0.707±0.055	**0.872±0.032**	0.639±0.063	0	0.555±0.138	0.764±0.056	0.523±0.163
0.2	0.716±0.034	0.852±0.058	0.680±0.046	0.1	0.701±0.061	0.761±0.102	0.743±0.081
0.3	0.716±0.061	0.835±0.049	0.689±0.093	0.2	0.704±0.059	0.784±0.067	0.712±0.096
0.4	0.755±0.057	0.803±0.061	0.766±0.064	0.3	0.776±0.043	0.775±0.072	0.834±0.030
0.5	0.776±0.058	0.821±0.056	0.786±0.079	0.4	0.778±0.020	0.763±0.040	**0.850±0.031**
0.6	**0.793±0.035**	0.835±0.061	0.814±0.040	0.5	**0.814±0.027**	**0.829±0.046**	0.838±0.038
0.7	0.788±0.056	0.774±0.088	0.857±0.036	0.6	0.777±0.034	0.810±0.035	0.806±0.053
0.8	0.755±0.039	0.740±0.076	0.844±0.040	0.7	0.771±0.027	0.810±0.046	0.788±0.034
0.9	0.739±0.065	0.672±0.092	**0.902±0.049**	0.8	0.758±0.034	0.776±0.036	0.813±0.075
				0.9	0.735±0.036	0.743±0.112	0.824±0.091
				1	0.744±0.042	0.715±0.071	0.843±0.049
